# Short-term joint effects of ambient PM_2.5_ and O_3_ on mortality in Beijing, China

**DOI:** 10.3389/fpubh.2023.1232715

**Published:** 2023-08-07

**Authors:** Ying Zhang, Shaobo Zhang, Jinyuan Xin, Shigong Wang, Xiaonan He, Canjun Zheng, Shihong Li

**Affiliations:** ^1^Plateau Atmosphere and Environment Key Laboratory of Sichuan Province, School of Atmospheric Sciences, Chengdu University of Information Technology, Chengdu, China; ^2^State Key Laboratory of Atmospheric Boundary Layer Physics and Atmospheric Chemistry, Institute of Atmospheric Physics, Chinese Academy of Sciences, Beijing, China; ^3^Beijing Anzhen Hospital, Capital Medical University, Beijing, China; ^4^Chinese Center for Disease Control and Prevention, National Institute for Communicable Disease Control and Prevention, Beijing, China; ^5^Department of Respiratory and Critical Care Medicine, Beijing Institute of Respiratory Medicine and Beijing Chao-Yang Hospital, Capital Medical University, Beijing, China

**Keywords:** PM_2.5_, O_3_, combined air pollution, joint effects, mortality, Beijing

## Abstract

**Introduction:**

In recent years, air pollution caused by co-occurring PM_2.5_ and O_3_, named combined air pollution (CAP), has been observed in Beijing, China, although the health effects of CAP on population mortality are unclear.

**Methods:**

We employed Poisson generalized additive models (GAMs) to evaluate the individual and joint effects of PM_2.5_ and O_3_ on mortality (nonaccidental, respiratory, and cardiovascular mortality) in Beijing, China, during the whole period (2014–2016) and the CAP period. Adverse health effects were assessed for percentage increases (%) in the three mortality categories with each 10-μg/m^3^ increase in PM_2.5_ and O_3_. The cumulative risk index (*CRI*) was adopted as a novel approach to quantify the joint effects.

**Results:**

The results suggested that both PM_2.5_ and O_3_ exhibited the greatest individual effects on the three mortality categories with cumulative lag day 01. Increases in the nonaccidental, cardiovascular, and respiratory mortality categories were 0.32%, 0.36%, and 0.43% for PM_2.5_ (lag day 01) and 0.22%, 0.37%, and 0.25% for O_3_ (lag day 01), respectively. There were remarkably synergistic interactions between PM_2.5_ and O_3_ on the three mortality categories. The study showed that the combined effects of PM_2.5_ and O_3_ on nonaccidental, cardiovascular, and respiratory mortality were 0.34%, 0.43%, and 0.46%, respectively, during the whole period and 0.58%, 0.79%, and 0.75%, respectively, during the CAP period. Our findings suggest that combined exposure to PM_2.5_ and O_3_, particularly during CAP periods, could further exacerbate their single-pollutant health risks.

**Conclusion:**

These findings provide essential scientific evidence for the possible creation and implementation of environmental protection strategies by policymakers.

## 1. Introduction

Significant epidemiological research has shown that short-term exposure to ambient air pollution is substantially related to numerous detrimental health consequences (Fan et al., 2020; Stafoggia et al., 2022) (1). Among the various ambient air pollutants, particles with diameters ≤2.5 μm (PM_2.5_) and ozone (O_3_) are considered have serious dangerous to human health (2).

At one time, China, the world’s largest developing country, had the worst air pollution issue than other countries, which led to almost 2 million premature deaths annually (3). The Chinese government has implemented a variety of pollution prevention and control measures since 2013 to protect public health, including policy changes in energy, industrial, and transportation infrastructure (4). According to (5), there was a significant reduction of 30–50% in PM_2.5_ concentrations from 2013 to 2017. Despite this reduction, PM2.5 pollution episodes persist in China, especially in megacities (6, 7). Furthermore, the decreased PM_2.5_ also slow down the sink of hydroperoxy radicals and thus speeding up O_3_ production, resulting in the ground-level O_3_ levels in China have grown annually (8). Consequently, there was a cooccurrence of PM_2.5_ and O_3_ pollution (9–11). This cooccurrence is known as combined air pollution (CAP). CAP has received much interest in atmospheric environmental research (12, 13). However, the health risks caused by CAP are still unclear.

Given that humans are exposed to more than one air pollutant in real life, biological responses to inhaled pollutants likely depend on the interaction between individual pollutants (14). Ground-level O_3_ and PM_2.5_ are closely related and interact with each other, and thus they may have a combined negative impact on human body (15). Traditional time-series studies have focused on assessing health effects using single-pollutant models. Research on the combined health effects of multiple pollutants has been inadequate. In recent years, some new models and methods have been developed to simultaneously quantify the combined health effects of multiple pollutants. One such technique is the use of the cumulative risk index (*CRI*), which involves the linear combination of individual coefficients. This approach enables accurate estimation of cumulative effects, even in cases where there is a high correlation among variables (14), and has been recommended for joint estimates of multipollutant exposure effects on health outcomes (16). However, *CRI*-related studies are quite limited; most of these studies have been conducted in developed countries, and studies in developing countries are lacking (17, 18).

Beijing, as the capital of China, has a serious air pollution issue. CAP appears in Beijing from time to time, and the frequency continues to increase (19). It is still unknown how the CAP affects the health outcomes of a local population. Therefore, the goal of this manuscript was to evaluate the individual and combined effects of PM_2.5_ and O_3_ on nonaccidental and cause-specific mortality in Beijing, China, across the entire time period and during the CAP period, respectively. The joint health effects of PM_2.5_ and O_3_ were estimated by using the *CRI* index.

## 2. Data and methods

### 2.1. Health data

In this study, we collected data on the daily death counts in Beijing from January 1, 2014, to December 31, 2016, from the Chinese Center for Disease Control and Prevention (CDC). To classify the causes of death, we used the International Classification of Diseases, Tenth Revision (ICD-10). Nonaccidental causes, cardiovascular diseases, and respiratory diseases were categorized as A00-R99, I00-I99, and J00-J99, respectively.

### 2.2. Environmental data

PM_2.5_ and O_3_ data were retrieved from the China National Environmental Monitoring Center. The maximal 8-h average ozone concentration was selected as the O_3_ concentration metric according to World Health Organization (WHO) recommendations (20). PM_2.5_ and O_3_ concentrations were recorded hourly at 12 stationary monitoring sites (Olympic Sports Center, Dongsi, Changping, Tiantan, Guanyuan, Shunyi, Huairou, Dingling, Agriculture Exhibition Hall, Haidian, Wanshou Temple, and Gucheng) in Beijing. We first calculated the mean of the hourly PM_2.5_ and O_3_ concentrations from all 12 monitoring sites and then calculated the 24-h mean PM_2.5_ and daily maximal 8-h average ozone concentrations. Details of the PM_2.5_ and O_3_ concentration data collection methods can be found in our published articles (21). According to the Ministry of Ecology and Environment of China’s national Ambient Air Quality Standards released in 2012 (22), PM_2.5_ pollution levels are defined as daily average PM_2.5_ concentrations >75 μg·m^−3^, and O_3_ pollution levels are defined as daily average O_3_ concentrations >160 μg·m^−3^. As a result, CAP days were designated as days when both O_3_ and PM_2.5_ values were above the criterion for co-occurring air pollution, with O_3_ concentrations >160 μg·m^−3^ and PM_2.5_ concentrations >75 μg·m^−3^. In addition, we collected data on some meteorological factors, including the daily average surface temperature (°C) and relative humidity (*RH*) (%), which were retrieved from the China Meteorological Data Sharing Service System.^1^

### 2.3. Statistical methods

We employed four parallel time-series Poisson generalized additive models (GAMs) to evaluate the individual and joint effects of O_3_ and PM_2.5_ on nonaccidental, cardiovascular, and respiratory mortality during the whole period and the CAP period. These models include a single-pollutant model, multipollutant model, nonparametric bivariate response surface model, and stratification model.

First, we utilized the single-pollutant model as the basis to assess the individual effects of a single pollutant on health outcomes at different lag days, including single (lag days 0 and 1) and cumulative (lag days 01 and 04) effects. The following is an expression for Model 1:


(1) $$\begin{array}{l} \log\left[E\left(Y_{t}\right)\right]=\alpha +NS\left(Time,3*6/\text{year}\right)+NS\left(Temp,3\right)+\\ \;\;\;\;\;\;\;\;\;\;\;\;\;\;\;\;\;\;\;\;\;\;NS\left(RH,3\right)+\text{as}.\text{factor}\left(DOW\right)+\\ \;\;\;\;\;\;\;\;\;\;\;\;\;\;\;\;\;\;\;\;\;\;\;\text{as}.\text{factor}\left(\text{Holiday}\right)+\beta_{kt}x_{kt}=\beta_{kt}x_{kt}+COVs\; \end{array}$$

where *Y*_t_ and E(*Y*_t_) signify the daily death counts and predicted death counts on day *t*, respectively. α refers to the intercept. NS() is the natural cubic spline function. According to the minimum Akaike information criterion (AIC), *Time* with the degrees of freedom (*df*) 6/year was selected to control for secular trends, and the *df* of the daily mean temperature (Temp) and *RH* are both 3. *DOW* and *Holiday* are two dummy variables that indicate weekday and public holidays, respectively (23). $x_{kt}$ and $\beta_{kt}$ denote the specific air pollutant concentrations and the corresponding coefficient on day *t*, respectively. Additionally, *COVs* represent all covariates including time, mean temperature, relative humidity, weekday, public holidays, and the intercept, respectively.

On this basis, we utilized a multipollutant model to evaluate the joint effects of PM_2.5_ and O_3_ on health outcomes at different lag days. The *CRI*, which was developed using estimates from multipollutant models, was used to assess the joint effects of multipollutant exposures (24). The multipollutant model and the formula for the *CRI* can be expressed as follows:


(2) $$\log\left[E\left(Y_{t}\right)\right]=\sum_{k=1}^{p}\beta_{kt}x_{kt}+COVs$$

(3) $$CRI_{t}=\exp\left(\sum_{k=1}^{p}\beta_{kt}*10\right)$$

where $x_{kt}$ and $\beta_{kt}$ denote the specific air pollutant concentrations and the corresponding coefficient on day *t*, respectively. The *COVs* are identical to those in Model (1). p indicates the type of air pollutant. *CRI_t_* denotes the joint effects of *p* air pollutant mixtures on day *t*.

The *CRIs* obtained from the multipollutant models were compared with the effect estimates of the single-pollutant models. If the effect estimate from the single-pollutant model was as high as the *CRI* from the multipollutant model, it indicated that the influence of only one pollutant was adequate to reflect the total pollutant mixture and that there were no synergistic effects.

Third, we also used a nonparametric bivariate response surface model to intuitively analyze the combined effects of PM_2.5_ and O_3_ on health outcomes. The model can be expressed as follows:


(4) $$\log\left[E\left(Y_{t}|X\right)\right]=ST\left(PM_{2.5},O_{3}\right)+COVs$$

ST() denotes the cubic regression splines. The *COVs* are identical to those in Model (1).

Fourth, the pollutant stratification model was employed to quantitatively assess the joint effects of PM_2.5_ and O_3_ on health outcomes during the CAP period. The model can be expressed as follows:


(5) $$\log\left[E\left(Y_{t}|X\right)\right]=m\beta_{it}O_{3}+m\beta_{jt}PM_{2.5}+COVs$$

where *m* is an indicator variable that is used to represent the CAP days. *m* = 1 represents co-occurring air pollution of PM_2.5_ and O_3_; otherwise, *m* = 0. *β*_it_ and *β*_jt_ represent the coefficients of O_3_ and PM_2.5_ on day t, respectively. The *COVs* are the same as those in Model (1).

To evaluate the models’ robustness, several sensitivity studies were carried out. We changed the *df* of *Time* from 7 to 10 per year and the *df* of mean temperature and *RH* from 3 to 5 for the single-pollutant model.

R 4.2.3 software with the “mgcv” package was used for all analyzes. For each 10-μg/m^3^ increase in PM_2.5_ and O_3_, the estimated individual and joint effects are shown as percentage changes (%) along with 95% confidence intervals (95% CIs).

## 3. Results

Table 1 summarizes the environmental and mortality data in Beijing, China, from 2014 to 2016. On average, there were 146 nonaccidental deaths per day, of which 64 were due to cardiovascular diseases and 17 were due to respiratory diseases. The annual mean temperature and *RH* were 15.65°C and 53%, respectively. Additionally, the annual average concentrations of PM_2.5_ and O_3_ were 78.97 and 118.10 μg/m^3^, respectively. Based on the statistical analysis, the daily average PM_2.5_ and O_3_ concentrations exceeded the threshold set by the Air Quality II Guidelines (75 μg·m^−3^ for PM_2.5_ and 160 μg·m^−3^ for O_3_, respectively) on 322 and 280 days, respectively (22). There were 59 CAP days during the study period, indicating serious air pollution in Beijing, China.

**Table 1:** Daily summary statistics of the air pollution levels, meteorological variables and number of deaths in Beijing, China, from 2014 to 2016.

Variables	Daily measures						No. of days
	Mean	Minimum	1st Q	Median	3rd Q	Maximum	
Deaths (*n*)							
Nonaccidental	146 ± 22	95	130	142	158	242	1,096
Cardiovascular	64 ± 14	33	55	62	72	125	1,096
Respiratory	17 ± 6	4	13	16	20	38	1,096
Environment variables							
Mean temperature (°C)	15.65 ± 10.97	−14.30	3.00	15.65	24.10	32.60	1,096
Relative humidity (%)	53.00 ± 20.03	8.00	37.00	53.00	69.00	99.00	1,096
PM_2.5_ (μg/m^3^)	78.97 ± 70.41	7.68	30.00	59.73	104.44	477.43	1,096
O_3_ (μg/m^3^)	118.10 ± 72.17	2.00	60.70	100.10	170.20	348.10	1,096

1st Q, first quartile; 3rd Q, third quartile.

**Figure 1:**
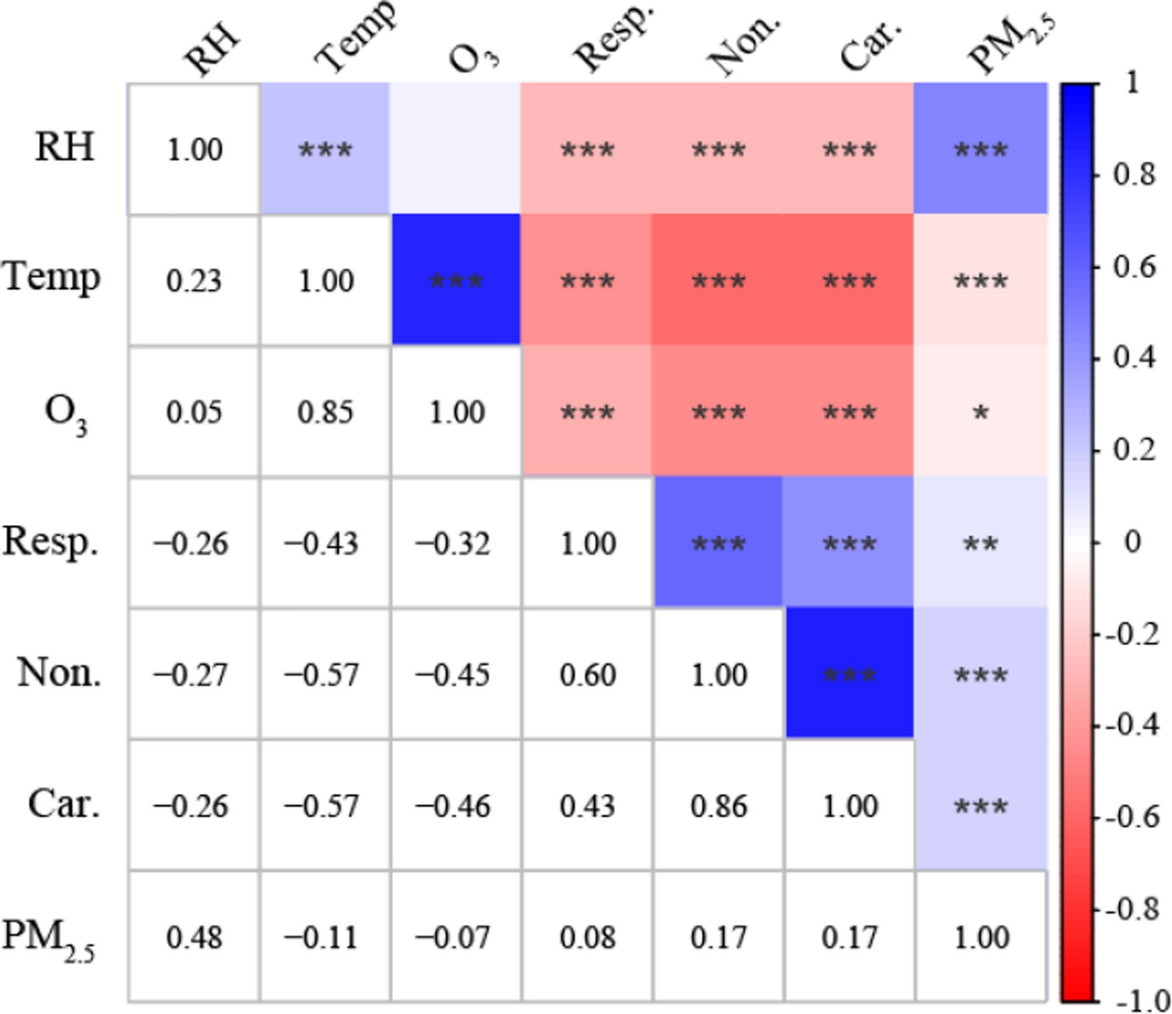
Spearman correlation matrix between the three mortality categories and different environmental factors in Beijing, China. *p < 0.05, **p < 0.01, ***p < 0.001. Non, Nonaccidental mortality; Car, Cardiovascular mortality; Resp, Respiratory mortality; Temp, mean temperature; RH, relative humidity.

The Spearman correlation coefficients of the three mortality categories and different environmental factors are shown in Figure 1. The three mortality categories were all significantly negatively correlated with the mean temperature, *RH*, and O_3_ concentration and significantly positively correlated with the PM_2.5_ concentration. The Spearman correlation between PM_2.5_ and O_3_ was low even though it was statistically significant (*r* = −0.07, *p* < 0.001), indicating the possibility of interaction effects on three mortality categories.

**Figure 2:**
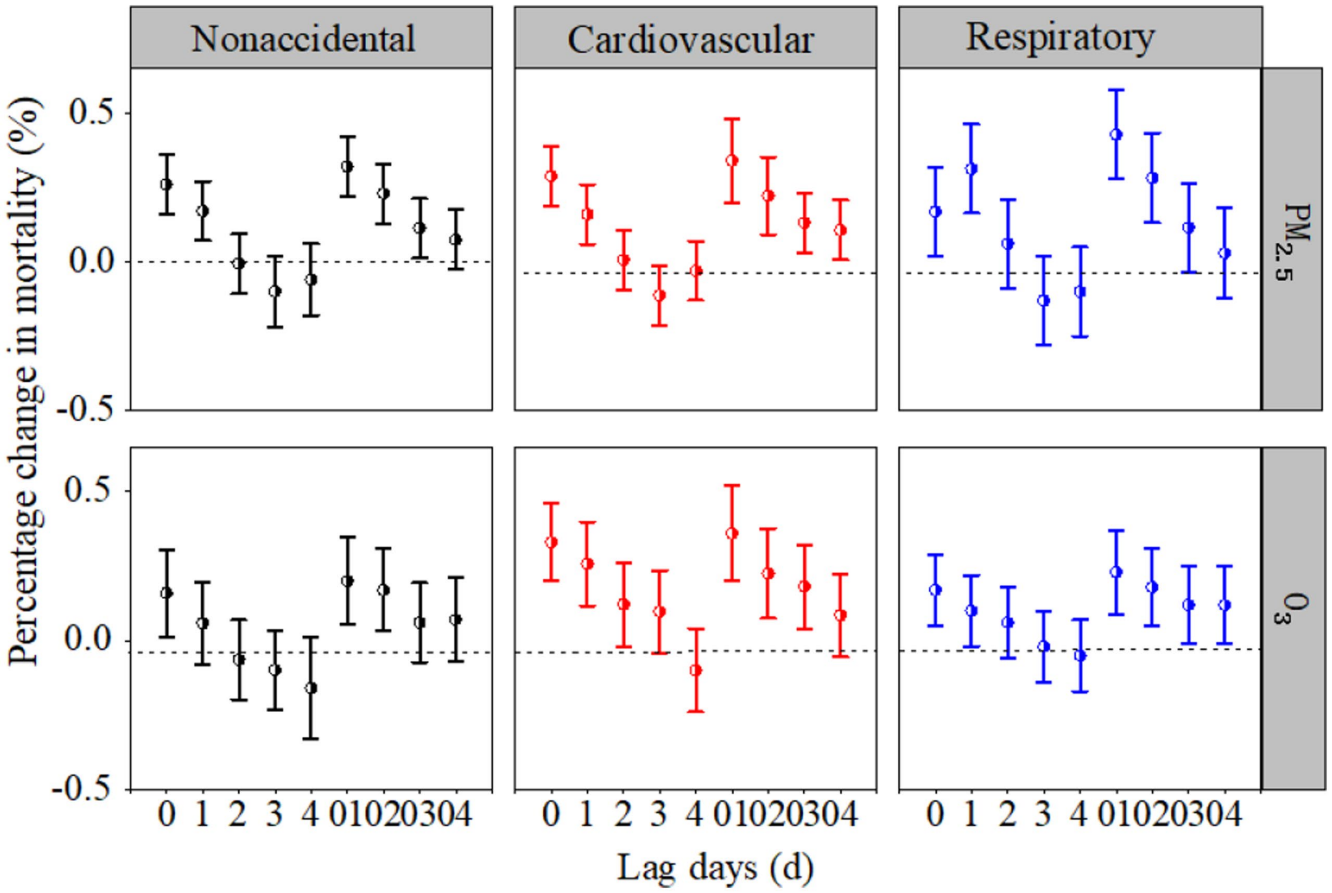
Percentage changes (%) in the three mortality categories associated with each 10-μg/m^3^ increase in PM_2.5_ and O_3_ concentrations at different lag days in the single-pollutant models.

Figure 2 illustrates the individual effects of PM_2.5_ and O_3_ on health outcomes at different lags. The individual effects of PM_2.5_ and O_3_ on the three mortality categories all peaked at lag day 01. Specifically, the increase in the nonaccidental, cardiovascular, and respiratory mortality categories was 0.32% (95% CI: 0.21, 0.43%), 0.36% (95% CI: 0.21, 0.50%), and 0.43% (95% CI: 0.28, 0.58%) for each 10-μm^−3^ increase in the PM_2.5_ concentration (lag day 01), and 0.22% (95% CI: 0.08, 0.36%), 0.37% (95% CI: 0.21, 0.53%), and 0.25% (95% CI: 0.12, 0.37%) for each 10-μg/m^3^ increase in the O_3_ concentration (lag day 01), respectively.

**Figure 3:**
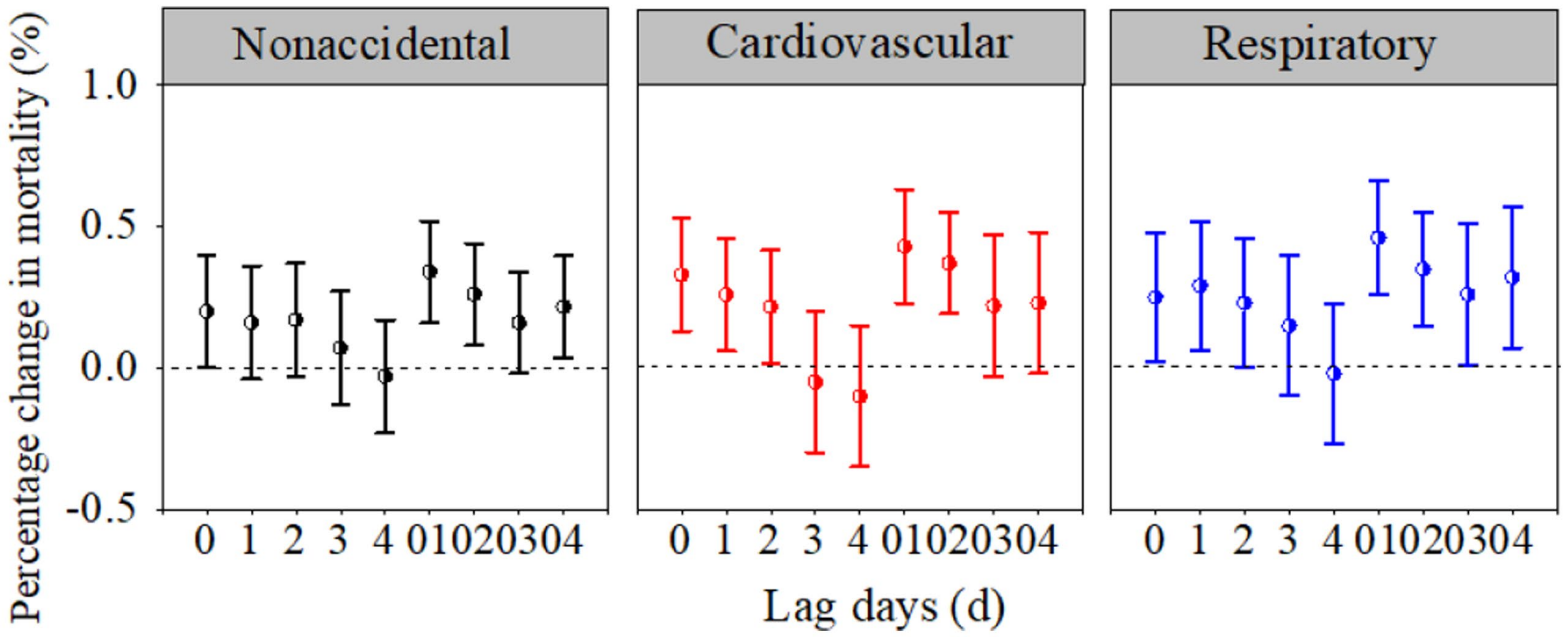
Percentage changes (%) in the three mortality categories associated with each 10-μg/m^3^ increase in the PM_2.5_ and O_3_ concentrations at different lag days in the multipollutant models.

Figure 3 depicts the joint effects of PM_2.5_ and O_3_ on health outcomes at different lags. As with the individual effects of PM_2.5_ and O_3_, the joint effects of PM_2.5_ and O_3_ on the three mortality categories all peaked at lag day 01. The corresponding *CRIs* for nonaccidental, respiratory and cardiovascular mortality were 0.34% (95% CI: 0.16, 0.52%), 0.43% (95% CI: 0.21, 0.65%), and 0.46% (95% CI: 0.23, 0.70%), respectively. Importantly, for the same category of diseases, the joint effect represented by *CRI* was higher than for any single pollutant effect estimate at lag day 01. Overall, the *CRI*s implied that a single-pollutant effect did not accurately represent the whole health effects of the mixture. In the subsequent analysis, both PM_2.5_ and O_3_ at lag day 01 were used as the research objects.

**Figure 4:**
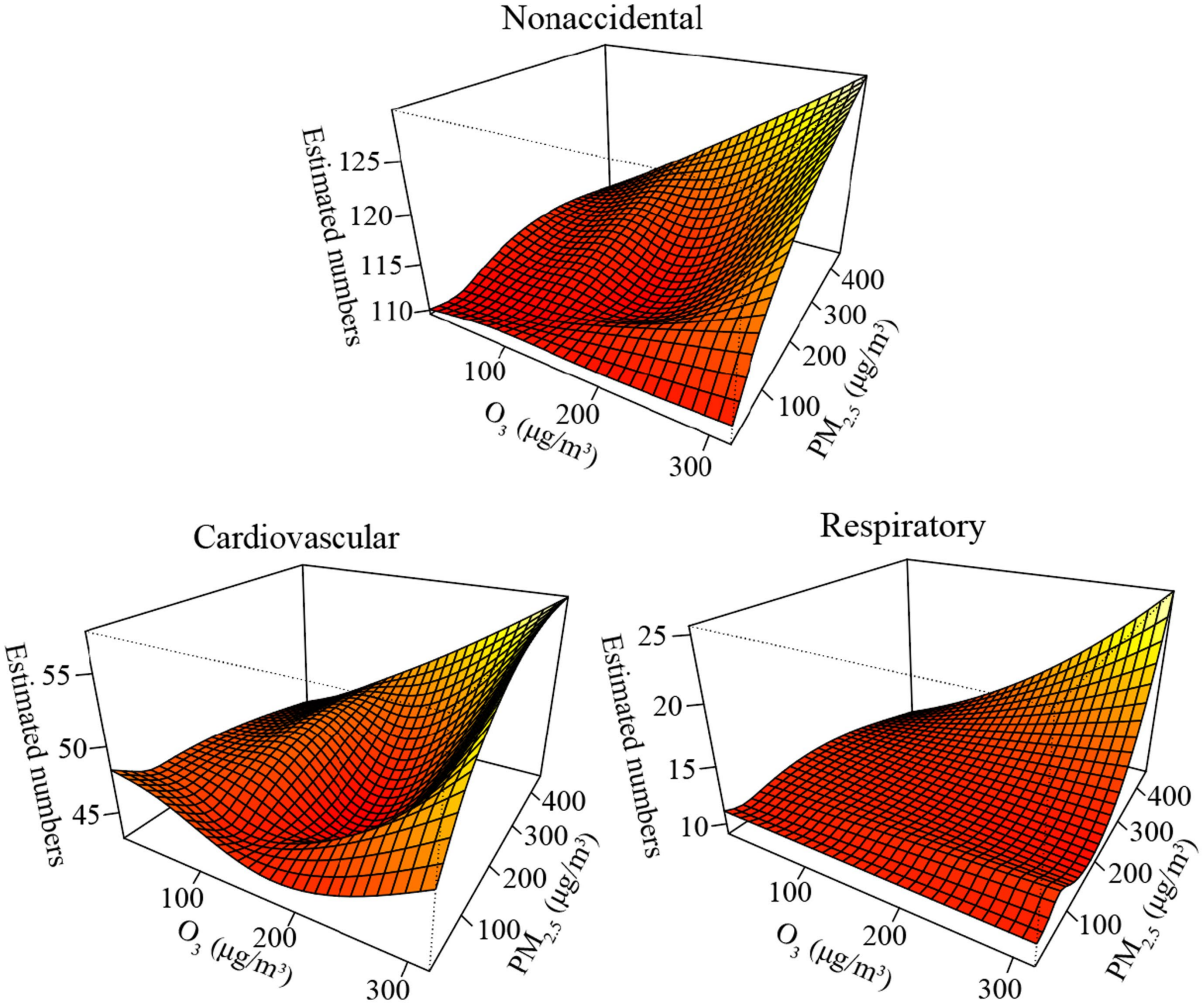
Bivariate response surfaces of PM_2.5_ and O_3_ for nonaccidental, cardiovascular, and respiratory deaths in Beijing, China.

Figure 4 illustrates the combined effects of PM_2.5_ and O_3_ on the three mortality categories using three-dimensional visualization graphs. The response surfaces show that the combined effects of PM_2.5_ (lag day 01) and O_3_ (lag day 01) on nonaccidental, cardiovascular, and respiratory deaths were complicated. Notably, when high concentrations of PM_2.5_ and O_3_ coexisted, all three categories (nonaccidental, cardiovascular, and respiratory fatalities) reached their maximums, showing that the interaction effects could be synergistic.

Table 2 depicts the individual and joint effects of PM_2.5_ (lag day 01) and O_3_ (lag day 01) on health outcomes during the whole period and the CAP period. For the same kind of illness, the *CRIs* of the joint effects during both the whole period and the CAP period were higher than any single-pollutant effect estimates. In addition, the joint effects during the CAP period were remarkably larger than those during the whole period, indicating that the CAP period further exacerbated the combined effects of PM_2.5_ and O_3_ on the three mortality categories.

**Table 2:** Percentage changes (%) in nonaccidental, respiratory, and cardiovascular mortality associated with each 10-μg/m^3^ increase in the PM_2.5_ and O_3_ concentrations during the whole period and the CAP period.

Air pollutants	Percentage change% (95% CI)		
	Nonaccidental	Cardiovascular	Respiratory
Single-pollutant models			
PM_2.5_^a^	0.32 (0.21, 0.43)*	0.36 (0.21, 0.52)*	0.43 (0.28, 0.58)*
O_3_^a^	0.22 (0.08, 0.36)*	0.37 (0.21, 0.53)*	0.25 (0.12, 0.37)*
PM_2.5_^b^	0.29 (0.17, 0.41)*	0.41 (0.12, 0.70)*	0.42 (0.18, 0.66)*
O_3_^b^	0.28 (0.08, 0.49)*	0.42 (0.23, 0.62)*	0.31 (0.15, 0.47)*
Multipollutant models			
O_3_ + PM_2.5_^a^	0.34 (0.16, 0.52)*	0.43 (0.21, 0.65)*	0.46 (0.23, 0.70)*
O_3_ + PM_2.5_^b^	0.58 (0.20, 0.96)*	0.79 (0.46, 1.12)*	0.75 (0.42, 1.08)*

a During the whole period.

b During the CAP period of co-occurring air pollution of PM_2.5_ and O_3_.*indicates *p* < 0.05.

According to the results of the sensitivity analyzes, the effects of O_3_ (or PM_2.5_) remained robust regardless of the change in the *df* of the time (see Supplementary Figure S1), the *df* of the mean temperature, and the *df* of the *RH* (see Supplementary Table S1).

## 4. Discussion

The CAP of PM_2.5_ and O_3_ has become a major environmental and health concern worldwide (7). Evaluating the short-term individual and joint effects of PM_2.5_ and O_3_ on health outcomes could provide valuable evidence for policymakers to regulate and prevent the accumulation of PM_2.5_ and O_3_. Our findings demonstrated that PM_2.5_/O_3_ was significantly associated with nonaccidental and cause-specific (cardiovascular and respiratory) mortality in Beijing, China. Additionally, the joint effects of the dual pollutants could further exacerbate their individual effects, especially during the CAP period.

Numerous studies of the individual effects of air pollutants, particularly PM_2.5_ and O_3_, on public health have been conducted (1, 25). For example, a meta-analysis conducted in 272 Chinese cities by Chen et al. (26) showed that a 10-μg/m^3^ increase in the PM_2.5_ concentration was associated with an increase in nonaccidental, cardiovascular, and respiratory mortality of 0.27, 0.39, and 0.29%, respectively. Another meta-analysis in China (27) revealed that an increase of 10-μg/m^3^ in the O_3_ concentration caused increases of 0.24 and 0.27% in nonaccidental and cardiovascular mortality, respectively. In this study, the results from the single-pollutant models revealed that each 10-μg/m^3^ increase in the PM_2.5_ concentration caused increases of 0.32, 0.36, and 0.43% in nonaccidental, cardiovascular, and respiratory mortality, respectively, and each 10-μg/m^3^ increase in the O_3_ concentration caused increases of 0.22, 0.37, and 0.25% in nonaccidental, cardiovascular, and respiratory mortality, respectively, in Beijing, China. Our estimates of the PM_2.5_–mortality and O_3_–mortality relationships were generally consistent with those of previous studies.

In the multipollutant models, our findings suggested that the estimates of the joint effects of the two air pollutants on mortality were higher than those for any individual effect for the same kind of illness. Consistent with our findings, a study conducted by Lei et al. (28) in Hefei, China, indicated that the effects of the health risks caused by PM_2.5_ on nonaccidental mortality increased when O_3_ was included, and vice versa, indicating that O_3_ and PM_2.5_ could aggravate each other’s unfavorable health effects. A cross-sectional study conducted in six countries revealed a synergistic interaction effect of PM_2.5_ and O_3_ on disease deterioration (29). However, in contrast to our findings, Qu et al. (30) observed that when O_3_ was included, the effect of PM_2.5_ on nonaccidental mortality was reduced. Moreover, several earlier studies showed no interaction effects of PM_2.5_ and O_3_ (31, 32). This inconsistency could be attributed to differences in the chemical composition, source, and toxicity of PM_2.5_ and O_3_ in different regions. Furthermore, the differences in study methods and individual sensitivity to pollutants can also lead to different results (33).

Notably, the patterns of the combined effects of PM_2.5_ and O_3_ on mortality demonstrated that coexisting high concentrations of PM_2.5_ and O_3_ could have synergistic effects on three mortality categories (34). Biological mechanisms have been somewhat postulated to explain the potential interaction effect of PM_2.5_ and O_3_ pollution on respiratory and cardiovascular mortality, despite the lack of clear evidence for a direct synergistic effect of the two pollutants on illnesses. For example, a few toxicology experiments on rats validated that the particulate matter served as a carrier for O_3_, delivering O_3_ into the body (35). Inhaling particles and O_3_ together had a synergistic impact on airway responsiveness and allergic inflammation in mice (36), suggesting that combined exposure to O_3_ and PM_2.5_ markedly increased health risks (37). Therefore, people, especially those with chronic respiratory and cardiovascular diseases, should strengthen protection measures and reduce outdoor activities, especially on CAP of PM_2.5_ and O_3_ days.

The key advantage of this study is as follows: Current research on CAP primarily focuses on the characteristics of changes in PM_2.5_ and O_3_ concentrations, meteorological causes, and their mutual influences. However, there is less emphasis on the joint health effects of PM_2.5_ and O_3_ during CAP periods (7, 38). Furthermore, traditional multipollutant models mainly focus on describing the difference in the health effects of a single pollutant before and after the addition of other pollutants without quantifying the combined effects of multiple pollutants (6). Our study differs from traditional studies, as we utilized multiple methods to examine the harmful health effects associated with exposure to one and two pollutants. We also conducted stratification studies on pollution, with a specific focus on the combined health effects of PM_2.5_ and O_3_ during the CAP period. Furthermore, we used the *CRI* to accurately quantify the joint effects of PM_2.5_ and O_3_ during both the whole and CAP periods. This approach addresses the limitations of previous research to a significant extent (16).

There are several limitations of our study that should be acknowledged. First and foremost, due to the difficulty in obtaining disease data in China, the study only included a 3-year disease death time series, and the time coverage was relatively limited. The latest year’s death data could not be obtained, which could reduce the statistical power. Second, in keeping with many previous studies (4, 39), we did not collect data on the real-time pollution exposure levels of individuals and only used the outdoor air pollutant concentration to represent individual PM_2.5_ and O_3_ exposure levels, which inevitably led to some deviation in the results (33). Third, the two most dangerous pollutants in China at this time are PM_2.5_ and O_3_. This study only tentatively carried out research on the interaction effect between PM_2.5_ and O_3_ on public health and did not carry out in-depth research on interaction effects with other air pollutants (such as O_3_ and nitrogen dioxide, sulfur dioxide and PM_2.5_). Therefore, with the improvement of research methods at a later stage, further in-depth study of the health effects of interactions between different air pollutants on human health should be carried out.

## 5. Conclusion

Our findings showed that exposure to PM_2.5_ and O_3_ may be significant risk factors for nonaccidental, cardiovascular, and respiratory mortality in Beijing, China. Moreover, we found that combined exposure to PM_2.5_ and O_3_ could amplify their individual effects on three mortality categories, particularly during CAP of PM_2.5_ and O_3_ periods. Therefore, during the CAP periods, the public should take timely preventive measures and reduce outdoor activities to some extent to reduce air pollution hazards.

## Data availability statement

The data analyzed in this study is subject to the following licenses/restrictions: Authors are not allowed to disclose data. Requests to access these datasets should be directed to YZ, zhangy881208@126.com.

## Author contributions

YZ: writing—review and editing, methodology, designed the research, and wrote the manuscript. SZ and XH: methodology and designed the research. JX: methodology, designed and reviewed the research, and reviewed the research. SW: formal analysis and reviewed the research. CZ: collected and analyzed the data. SL: writing—review and editing, formal analysis, and collected and analyzed the data. All authors contributed to the article and approved the submitted version.

## Funding

This study was supported by the National Nature Science Foundation of China (42005136), Innovation Team Fund of Southwest Regional Meteorological Center, China Meteorological Administration (XNQYCXTD-202203), China Postdoctoral Science Foundation (2020M670419), and National Key Research and Development Project Program of China (2016YFA0602004). This work was supported in part by Chengdu Plain Urban Meteorology and Environment Sichuan Provincial Field Scientific Observation and Research Station, Chengdu, China.

## Conflict of interest

The authors declare that the research was conducted in the absence of any commercial or financial relationships that could be construed as a potential conflict of interest.

## Publisher’s note

All claims expressed in this article are solely those of the authors and do not necessarily represent those of their affiliated organizations, or those of the publisher, the editors and the reviewers. Any product that may be evaluated in this article, or claim that may be made by its manufacturer, is not guaranteed or endorsed by the publisher.

## Footnotes

^1^ http://data.cma.cn/
