# Supplementary material for: Short-term joint effects of ambient PM2.5 and O3 on mortality in Beijing, China
Source: Front Public Health. 2023 Aug 7;11:1232715. doi: 10.3389/fpubh.2023.1232715 (PMC10441666; doi:10.3389/fpubh.2023.1232715)
Supplement: Supplementary file 1 [file Data_Sheet_1.docx]

**Supplementary Materials**

***CRI* Details:**

The formula of the *CRI* was: exp ($\beta^{'}x$), where $\beta$ is a *p* vector of parameter estimates and *x* is a *p* vector of air pollutants concentrations, then $\beta^{'}x$ was: $\beta_{1}x_{1}$*+…+* $\beta_{p}x_{p}$ for *p* air pollutants. The 95% confidence interval (95% CI) of *CRI* was defined by: *exp*{$\beta^{'}x$± 1.96$\sqrt{(x'Cx)}$}, where *C* is the *p* by *p* covariance matrix of the estimates $\beta$.


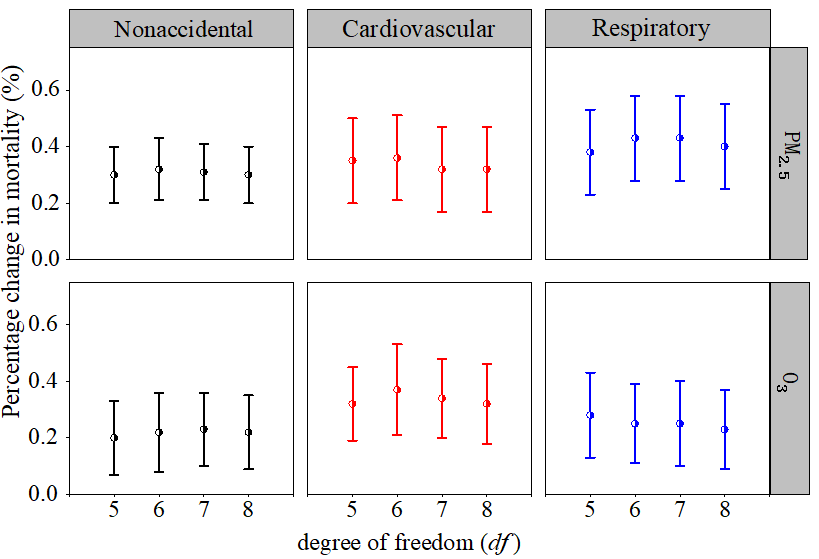


**Fig. S1** Sensitivity analysis of the PM_2.5_ (or O_3_)-mortality association using different *df* for Time in Beijing, China in single-pollutant models

**Table S1** Sensitivity analysis of the PM_2.5_ (or O_3_)-mortality association using different *df* for average temperature and relative humidity in Beijing, China.

| Different *df*s | Mortality | Percentage changes% (95% confidence intervals (CI)) | |
| --- | --- | --- | --- |
|  |  | PM_2.5_ | O_3_ |
| Temp (*df* =3) | Nonaccidental | 0.32% (95% CI: 0.21%, 0.43%) | 0.22% (95% CI: 0.08%, 0.36%) |
| Temp (*df* =4) | Nonaccidental | 0.30% (95% CI: 0.18%, 0.42%) | 0.21% (95%CI: 0.06%, 0.35%) |
| Temp (*df* =5) | Nonaccidental | 0.31% (95%CI: 0.18%, 0.44%) | 0.17% (95%CI: 0.03%, 0.31%) |
| Temp (*df* =3) | Cardiovascular | 0.36% (95% CI: 0.21%, 0.50%) | 0.37% (95% CI: 0.21%, 0.53%) |
| Temp (*df* =4) | Cardiovascular | 0.34% (95%CI: 0.18%, 0.50%) | 0.35% (95%CI: 0.20%, 0.51%) |
| Temp (*df* =5) | Cardiovascular | 0.32% (95%CI: 0.15%, 0.49%) | 0.33% (95%CI: 0.17%, 0.49%) |
| Temp (*df* =3) | Respiratory | 0.43% (95% CI: 0.28%, 0.58%) | 0.25% (95% CI: 0.12%, 0.37%) |
| Temp (*df* =4) | Respiratory | 0.44% (95%CI: 0.28%, 0.60%) | 0.23% (95%CI: 0.10%, 0.36%) |
| Temp (*df* =5) | Respiratory | 0.38% (95%CI: 0.21%, 0.55%) | 0.22% (95%CI: 0.08%, 0.35%) |
| *RH* (*df* =3) | Nonaccidental | 0.32% (95% CI: 0.21%, 0.43%) | 0.22% (95% CI: 0.08%, 0.36%) |
| *RH* (*df* =4) | Nonaccidental | 0.32% (95% CI: 0.21%, 0.43%) | 0.20% (95% CI: 0.07%, 0.33%) |
| *RH* (*df* =5) | Nonaccidental | 0.33% (95% CI: 0.24%, 0.42%) | 0.20% (95% CI: 0.08%, 0.32%) |
| *RH* (*df* =3) | Cardiovascular | 0.36% (95% CI: 0.21%, 0.50%) | 0.37% (95% CI: 0.19%, 0.55%) |
| *RH* (*df* =4) | Cardiovascular | 0.36% (95%CI: 0.21%, 0.50%) | 0.36% (95% CI: 0.18 %, 0.54%) |
| *RH* (*df* =5) | Cardiovascular | 0.35% (95%CI: 0.20%, 0.51%) | 0.35% (95%CI: 0.20%, 0.50%) |
| *RH* (df =3) | Respiratory | 0.43% (95% CI: 0.28%, 0.58%) | 0.25% (95% CI: 0.12%, 0.37%) |
| *RH* (df =4) | Respiratory | 0.43% (95% CI: 0.28%, 0.58%) | 0.25% (95% CI: 0.13%, 0.37%) |
| *RH* (df =5) | Respiratory | 0.42% (95% CI: 0.27%, 0.56%) | 0.24% (95% CI: 0.12%, 0.36%) |

Abbreviation: PM_2.5_: particulate matter with an aerodynamic diameter ≤2.5 μm; O_3_: the maximum 8h daily average ozone concentration; Temp: average temperature; RH: relative humidity.
